# Reduced mosquito survival in metal-roof houses may contribute to a decline in malaria transmission in sub-Saharan Africa

**DOI:** 10.1038/s41598-019-43816-0

**Published:** 2019-05-23

**Authors:** Steve W. Lindsay, Musa Jawara, Julia Mwesigwa, Jane Achan, Nabie Bayoh, John Bradley, Balla Kandeh, Matthew J. Kirby, Jakob Knudsen, Mike Macdonald, Margaret Pinder, Lucy S. Tusting, Dan J. Weiss, Anne L. Wilson, Umberto D’Alessandro

**Affiliations:** 1 Department of Biosciences, Durham University, Durham, DH13LE UK; 2 London School of Hygiene and Tropical Medicine, London, WC1E 7HT UK; 3 Medical Research Council Unit Gambia at the London School of Hygiene and Tropical Medicine, Fajara, The Gambia; 4 Adaptive Management and Research Consultants, Kisumu, Kenya; 5 National Malaria Control Programme, Banjul, The Gambia; 6 The Royal Danish Academy of Fine Arts, School of Architecture, Design and Conservation, The School of Architecture, Copenhagen, Denmark; 7 Independent Consultant, Arlington, USA; 8 Big Data Institute, Nuffield Department of Medicine, University of Oxford, Oxford, UK; 9 Department of Vector Biology, Liverpool School of Tropical Medicine, Liverpool, UK

**Keywords:** Ecological epidemiology, Malaria

## Abstract

In The Gambia, metal-roof houses were hotter during the day than thatched-roof houses. After 24 h, the mortality of *Anopheles gambiae*, the principal African malaria vector, was 38% higher in metal-roof houses than thatched ones. During the day, mosquitoes in metal-roof houses moved from the hot roof to cooler places near the floor, where the temperature was still high, reaching 35 °C. In laboratory studies, at 35 °C few mosquitoes survived 10 days, the minimum period required for malaria parasite development. Analysis of epidemiological data showed there was less malaria and lower vector survival rates in Gambian villages with a higher proportion of metal roofs. Our findings are consistent with the hypothesis that the indoor climate of metal-roof houses, with higher temperatures and lower humidity, reduces survivorship of indoor-resting mosquitoes and may have contributed to the observed reduction in malaria burden in parts of sub-Saharan Africa.

## Introduction

Between 2000 and 2015, malaria infections in sub-Saharan Africa dropped by half and clinical disease by 40%, mainly due to the massive deployment of long-lasting insecticidal nets (LLINs), indoor residual spraying (IRS) and prompt diagnosis and treatment with effective antimalarials^1^. These interventions, however, do not completely explain the reduction in malaria^1^, and in many places malaria started to decline before the large-scale implementation of malaria interventions^2–7^. This indicates that other factors, not just deliberate malaria control, are contributing to the decline in malaria.

Here we examine whether modern housing may be an environmental factor contributing to the decline in malaria transmission. Since the start of the millennium, Africa’s housing stock has changed rapidly and traditional thatched-roofe, mud-walled houses, once common in many parts of sub-Saharan Africa^8^, are being replaced by millions of metal-roof houses, with walls often constructed from cement blocks or burnt bricks. Between 2000 and 2015, improved housing has more than doubled in the region, excluding South Africa and desert areas, from 11% to 23%^9^. Such large changes in housing stock may have a profound influence on malaria transmission since there is growing evidence from observational studies that the malaria risk is lower for people living in modern houses than for those in traditional thatched-roof, mud-walled housing^10^. More recently, a multi-country analysis of 29 malaria surveys from 21 countries in sub-Saharan Africa found that modern housing was associated with lower odds of malaria infection and clinical malaria than traditional housing, with an effect size similar to LLINs^11^.

Metal-roof housing may reduce malaria transmission in at least two ways. First, individual protection may be provided by house designs typically associated with metal roofs. Metal-roof houses often have fewer mosquitoes than thatched ones since they are often constructed with the eaves closed, unlike thatched houses that traditionally have open eaves. This is important since the principal route by which *Anopheles gambiae* s.l., the major African vector of malaria, enters houses is through the open eaves. As such, closing eaves results in a threefold reduction in house entry^12^ and an associated decline in entomological inoculation rate. As ≥80% of malaria transmission occurs indoors^13,14^, reducing house entry by malaria mosquitoes likely reduces the risk of infection. Second, we propose the novel hypothesis that metal-roof houses may have a community effect, whereby vector survival declines because these houses are hotter than traditional thatched houses during the day^15^. After blood-feeding on a human host indoors, *An. gambiae* typically rest indoors for two to three days before leaving the house to locate an aquatic habitat in which to lay their eggs^16^. If the high temperatures associated with metal-roof houses reduce vector survival, it will reduce the proportion of the vector population that survive long enough to become infective. It is well known from the classic Ross-Macdonald malaria model that a reduction in vector survival has a disproportionately large impact on reducing vectorial capacity, a measure of malaria risk^17,18^.

We carried out a series of experiments to assess the impact of high temperatures on the behaviour and survival of *An. gambiae* s.l. in the field and laboratory. We also examined the association between metal-roof houses and malaria in The Gambia, a country that has experienced a gradual decline in malaria over the past 15 years^3,19^, but where in the eastern region, malaria remains stubbornly persistent despite intensive vector control over many years^20^. These studies allowed us to test, for the first time, the hypothesis that mosquito survival in metal-roof houses is lower than in thatched-roof houses.

## Results

### Mosquito survival in metal- and thatched-roof houses

**Figure 1:**
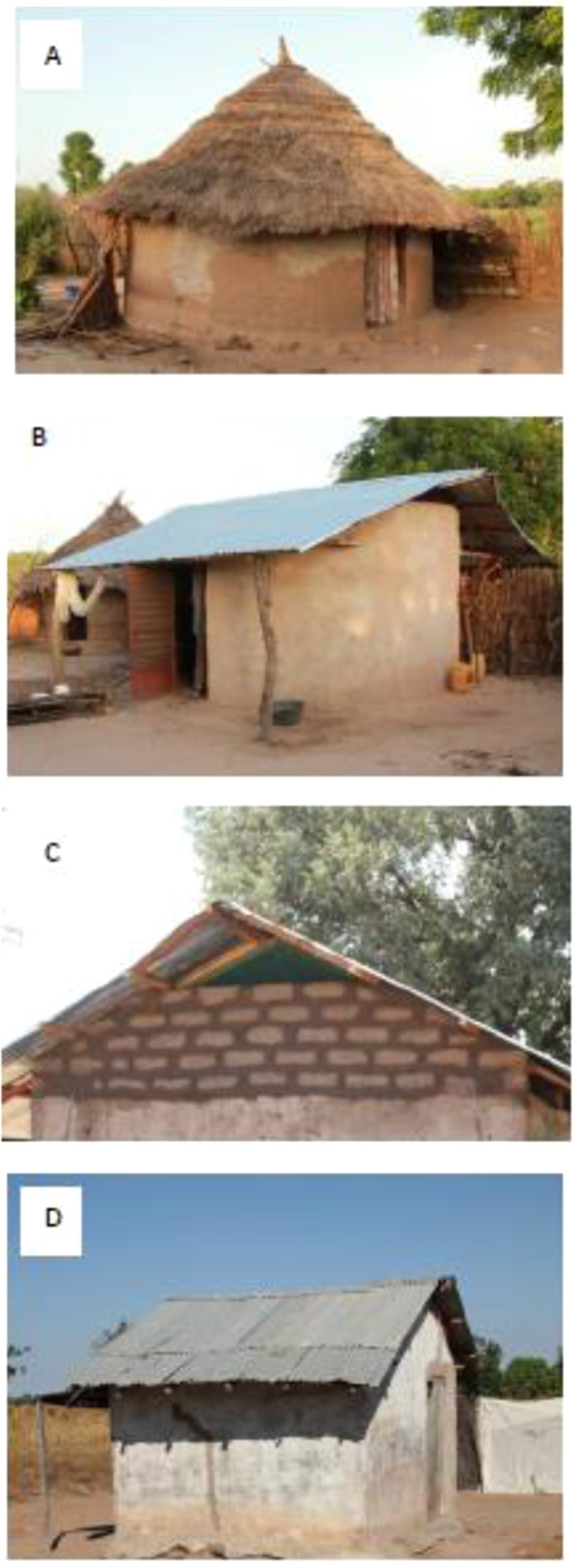
Types of study houses used in the mosquito survival experiments. Where (**A**) is a traditional thatched-roof house, (**B**) is a ventilated metal-roof house, (**C**) is a close-up of a screened window positioned in the gable ends of a ventilated house and (**D**) is a traditional metal roofed house without ventilation. Photographs by SW Lindsay.

We first carried out experiments to measure the temperature of metal-roofed and thatched-roof houses in rural Gambia and determine the mortality of caged blood-fed *An. gambiae* s.l. females during the rainy season, when malaria transmission is highest^21^. In 2016, we built and compared ventilated metal-roof houses (i.e. those with closed eaves, screened windows and doors), constructed for a randomised-controlled trial of housing against malaria^22^, with thatched-roof houses, the traditional housing typology in The Gambia. In 2017 we compared unventilated metal-roof houses with thatched-roof houses (Fig. 1). The ventilated house was a novel typology of housing which keeps the house cooler at night, than the typical unventilated metal-roof house found in Gambian villages^23^.

**Figure 2:**
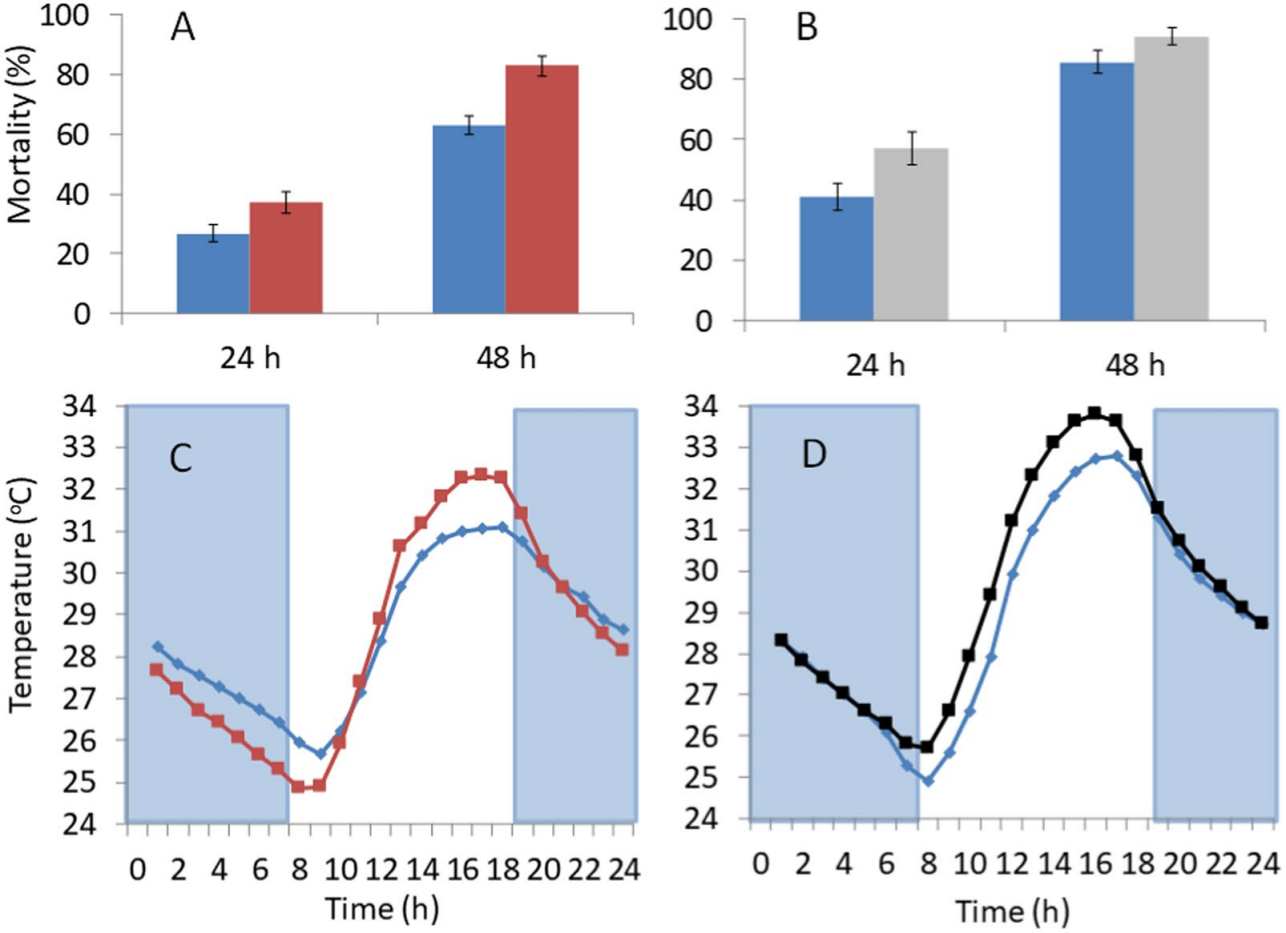
Survival of *An. coluzzii* in Gambian houses where (**A**) compares thatched-houses (blue) with ventilated metal-roof houses (red), (**B**) compares thatched-houses (blue) with unventilated metal-roofed houses (grey), (**C**) is indoor temperature in thatched-houses (blue) and ventilated metal-roofed houses (red) in 2016 and (**D**) is indoor temperature in thatched-houses (blue) and unventilated metal-roofed houses in 2017. Mortality after 48 h is a measure of mortality from 0 to 48 h. Error bars are standard errors. Blue shaded areas in (**C**,**D**) represents hours of darkness. In 2016, the mean indoor temperature in ventilated metal-roofed houses was temperature 29.7 °C (28.8–30.6 °C) and 28.7 °C (95% CIs = 28.0–29.4 °C) in thatched houses, whilst the maximum indoor temperature was 34.7 °C (95% CIs = 33.8–35.6 °C) in metal-roofed houses and 33.1 °C (95% CI = 32.1–34.1 °C) in thatched ones. In 2017, the mean indoor temperature in unventilated metal-roofed houses during the day was 30.3 °C (95% CIs = 29.5–31.1 °C) and 29.4 °C (28.7–30.1 °C) in thatched houses, whilst the maximum indoor temperature was 35.5 °C (95% CIs = 34.8–36.2) in unventilated metal-roofed houses and 34.5 °C (95% CI = 33.7–35.3 °C) in thatch-roofed houses.

Metal-roof houses were hotter during the day (06.30–18.30 h) than thatched houses. In 2016, the mean indoor temperature of ventilated metal-roof houses was 1.0 °C greater (95% CIs = 0.6–1.3 °C, paired t test, t = 5.473, n = 15, p < 0.001) than in thatched houses (Fig. 2), and the maximum indoor temperature was 1.5 °C greater (95% CIs = 1.0–2.1 °C, paired t test = 6.292, n = 15, P < 0.001). Similar results were found in 2017, where the mean indoor temperature in unventilated metal-roof houses was 0.9 °C greater (95% CIs = 0.6–1.2 °C, paired t test, t = 6.200, n = 15, p < 0.001) during the day than thatched houses (Fig. 2), whilst the maximum indoor temperature was 1.4 °C greater (95% CIs = 0.7–2.2 °C, paired t test = 4.031, n = 15, P = 0.001).

In both years, the mortality of blood-fed mosquitoes was higher in metal-roof than thatched houses. In 2016, the mean mortality of blood-fed mosquitoes after 24 h was 38.3% greater in ventilated metal-roof houses than thatch-roof houses (paired-t test, t = 3.565, df = 14, p = 0.003) and 31.4% greater after 48 h in metal-roof houses (paired -t test, t = 9.028, df = 14, p < 0.001; Fig. 2). In 2017, the mean mortality of blood-fed mosquitoes after 24 h was 38.5% higher in metal-roof houses than thatch-roof houses (paired-t test, t = 4.304, p = 0.01) and 9.9% higher after 48 h in metal-roof houses (paired -t test, t = 2.477, df = 14, p = 0.027). These findings suggest that the hotter temperatures experienced during the day in metal-roof houses, compared with traditional thatched houses, were associated with higher mosquito mortality.

### Indoor mosquito behaviour in metal-roof houses

We sought to better understand how wild mosquitoes responded to the changing temperatures in metal-roof houses during the day. During the last few weeks of the dry season 236 *An. gambiae* s.l. were collected, of which 75% were *An. coluzzii* and 25% *An. arabiensis*. At 07.00 h, 91.1% of the vectors were collected resting on the metal roof, but this declined to 24.0% at 14.00 h (paired-t test = 11.89, p < 0.001) as the roof warmed by an average of 10.1 °C, from 29.2 °C (95% CIs = 27.9–30.3 °C) to 39.3 °C (95% CIs = 37.9–40.9 °C; paired t test = 7.92, p = 0.01). Whilst the temperature of the floor and roof were similar at 07.00 h (paired t test = 0.32, p = 0.778), by 14.00 h the roof was 3.6 °C warmer than the floor (paired t test = 2.854, p = 0.046). These results suggest that mosquitoes moved away from extremely high temperatures, however they were still experiencing temperatures on the floor in the afternoon of 35.7 °C on average.

### Mosquito survival in the laboratory

**Figure 3:**
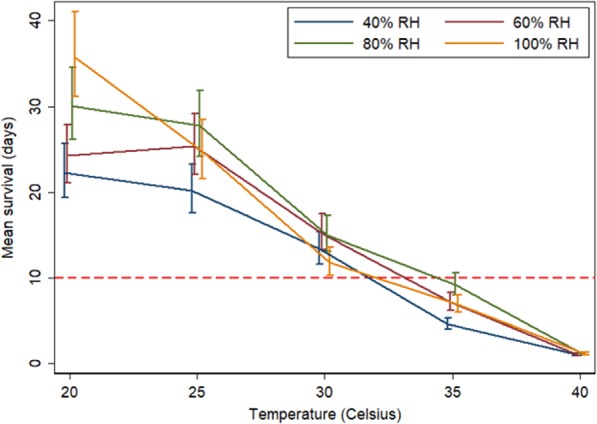
Survival of female *An. gambiae s.s*. maintained at different combinations of temperature and relative humidity (RH). The red broken line represents the minimum period for maturation of *P. falciparum* in the vector (10 days)^24^. Error bars are 95% confidence intervals. Rate ratios are shown in Supplementary Material (Table 2).

We assessed the effect of temperature and relative humidity on mosquito survival in the laboratory. We measured the daily survival of adult female *An. gambiae* s.s. held in cages at different combinations of temperature (20, 25, 30, 35 & 40 °C) and relative humidity (40, 60, 80 & 100%, Fig. 3). Daily mortality rate increased by 16.0% (95% CI = 15.4–16.6%) with a rise in temperature of 1 °C. Importantly, mortality rates doubled with an increase in temperature from 30 to 35 °C (Supplement Table 1), a temperature frequently recorded in Gambian houses (Fig. 2). At 35 °C few mosquitoes survived longer than 10 days, the minimum period required for a vector to become infectious^24^. A relative humidity of 40% also resulted in increased mortality compared with higher values. Malaria transmission in The Gambia is typically confined to the rainy season, or immediately after the end of the rainy season, when the relative humidity is high, above a mean of 70%^23,25^, but drops to a mean of 40% during the dry season, when there is little, if any, transmission^26^.

### Association between roof type and entomological and clinical outcomes

**Figure 4:**
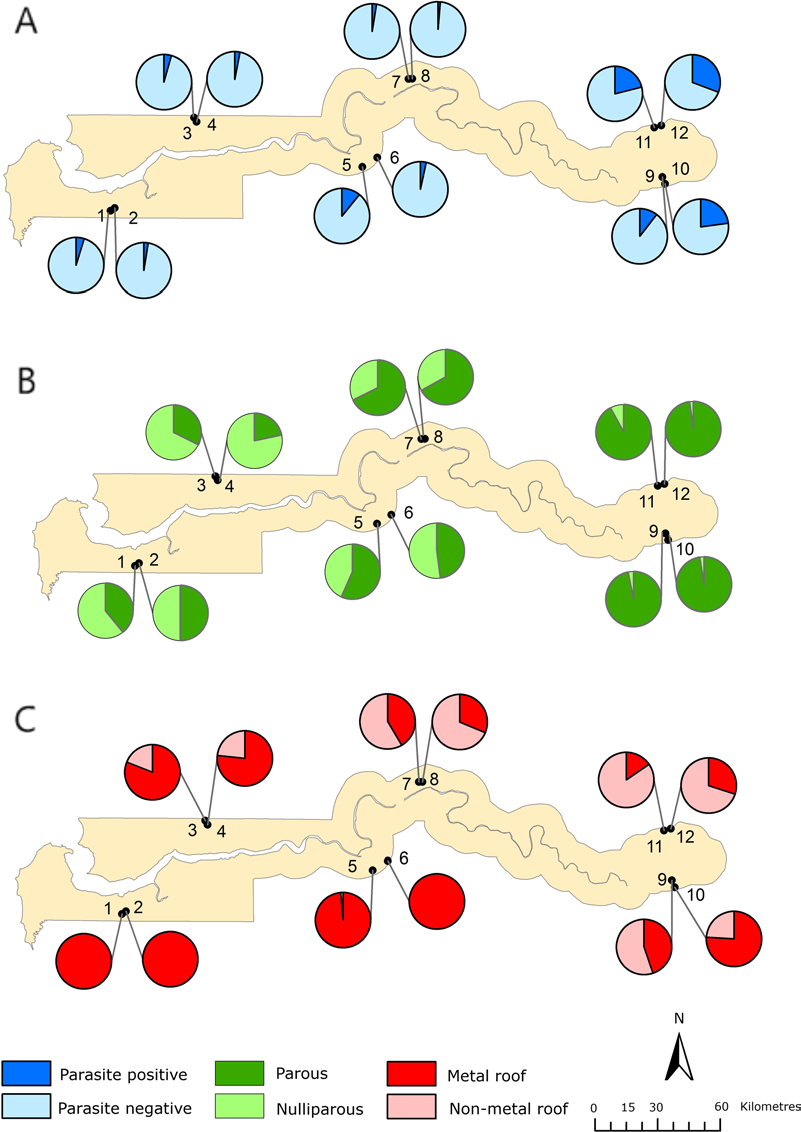
Distribution of (**A**) cumulative parasite prevalence in monthly surveys from June to December 2013 in children 6 months to 15 years old, (**B**) proportion of parous An. gambiae s.l., and (**C**) proportion of houses with metal roofs in 12 sites in The Gambia. Where 1 is Bessi, 2 is Ndemban Tenda, 3 is Chogen Wellingara, 4 is Yallal Ba, 5 is Dongoro Ba, 6 is Sinchu Njengudi, 7 is Ngeden, 8 is Sare Seedy, 9 is Njaiye, 10 is Madina Samak, 11 is Sare Wuro and 12 is Gunjur Koto. Non-metal roofs included thatched and tiled houses.

To explore the relationship between metal-roofed housing and malaria transmission, we carried out a secondary analysis of two studies in The Gambia; a nationwide one (Fig. 4) and one in eastern Gambia, where malaria transmission is higher.

In the nationwide study we demonstrated the association between entomological and clinical outcomes by showing that *Plasmodium falciparum* prevalence rates in children aged six months to fifteen years old in villages increased progressively with vectorial capacity (linear regression, r^2^ = 0.337, p = 0.028, Supplementary Material Table 2). Since vectorial capacity is most sensitive to changes in daily mosquito survival^17,18^, (Supplementary Material Table 1), we did an additional analysis to demonstrate that parity was also associated with parasite prevalence (linear regression, r^2^ = 0.535, p = 0.004). Because a reduction in parity (=decreased vector survival) is the mechanism by which we hypothesise metal roofs affect malaria transmission, hereafter we focused the analysis on identifying what environmental covariates were associated with parity. Higher parity values were associated with proportionately fewer metal roofed houses (linear regression, r^2^ = 0.375, p = 0.020, Fig. 4), warmer and more humid nights (linear regression with Mean Land Surface Temperature pm; r^2^ = 0.690, p = 0.001), and where the rainy season was concentrated in a shorter period (linear regression with SPP, r^2^ = 0.510, p = 0.005). There was no association between the other environmental variables (see methods) tested and parity. In the regional study, in which there was little difference in climate between villages, higher parity values were associated with a smaller proportion of metal-roof houses within villages (linear regression, r^2^ = 0.192, p = 0.007), but not with any of the climate covariates.

## Discussion

Our findings are consistent with the hypothesis that hotter metal-roof houses reduce vector survival when compared with traditional, cooler thatched-roof African houses. During the day, both ventilated and unventilated metal-roof houses were approximately 1.0 °C hotter, with maximum temperatures 1.5 °C hotter, than thatched houses. Importantly, mortality of blood-fed malaria vectors was 38% higher after 24 h in metal-roof houses than in traditional thatched-roof houses. The reason for increased mortality at elevated temperatures is unknown, although it is likely to be associated with the lethal effects of high temperatures on metabolism and higher levels of desiccation experienced by mosquitoes.

Our observations of wild mosquitoes in metal-roof houses indicate that adult females actively move away from extreme heat. At 07.00 h nearly all mosquitoes rested on the metal roof, but by 14.00 h as the roof was heated by the sun, they had moved to the cooler walls below, resting in dark corners and under beds and closer to humid water jars, at temperatures 3.6 °C less than the roof^27^. Critically, we found few mosquitoes resting on the metal roofs when temperatures approached 34 °C. Whilst we cannot ignore the possibility that movement of mosquitoes from the roof to the floor is dependent on time of day, as well as temperature, we think temperature plays a major role since in the laboratory mosquitoes actively move away from extremely high temperatures^28^. *Anopheles arabiensis* moves away from gradually increasing temperatures at 35.7 °C, whilst *An. gambiae s.s*., which is less heat tolerant, moved when temperatures reach 33.0 °C. These findings indicate behavioural avoidance of high temperatures by *An. gambiae* mosquitoes, presumably as a defence against the lethal impact of high temperatures. Since the maximum indoor temperature of metal-roof houses is around 35 °C for several hours each day during and immediately after the rainy season (when most malaria transmission occurs), the vector population in The Gambia may be near its climatic tolerance.

The natural movement of mosquitoes away from temperatures approaching 35 °C is likely to be an evolutionary-adaptive behaviour, increasing the survival of adult mosquitoes. This is supported by our laboratory studies showing that when *An. gambiae s.s*. adults are held at constant temperatures of 35 °C, few mosquitoes survive 10 days, the minimum period required after an infective blood meal for a mosquito to become infectious^24^. It is also likely that high temperatures in the field, besides being lethal to the vector, may also be lethal to the developing malaria parasites in the mosquito^26^. Recent laboratory work has confirmed the sterilising effect of high temperatures on malaria parasites, where malaria-infected *An. gambiae s.s*. maintained at 33 °C failed to produce sporozoites, the malaria parasite infectious stage^29^. A model of malaria force of infection, based on laboratory studies of *P. falciparum*-infected *An. stephensi*, showed that maximum transmission occurred at 26 °C, but dropped sharply over 30 °C, with no transmission at 35 °C, again confirming the lethality of temperatures over 30 °C^30^.

If our hypothesis that metal-roof houses are inimical to the survival of indoor-resting vectors, what does this imply for the increasing trend of building metal-roof houses across sub-Saharan Africa? In a nationwide survey in The Gambia, the survival of *An. gambiae* populations in a village, assessed by the proportion of parous mosquitoes (those that had laid at least one batch of eggs), declined as the proportion of houses with metal-roofs increased. This association also held in the far east of the country where malaria prevalence is the highest. Both findings support the hypothesis that metal-roof houses used on a large-scale could have a mass-killing effect, reducing the survival of malaria mosquitoes and thus malaria transmission. The importance of vector survival on malaria transmission is critical and is best illustrated using the Ross-Macdonald model^17,18^ where it can be demonstrated that small changes in daily vector survival, estimated from parity, resulted in large changes in vectorial capacity. In the nationwide study the importance of parity was also illustrated by the high malaria parasite rates in children found in sites with vector populations that had a high proportion of parous females.

Despite being a small country, The Gambia extends 338 km from the coast in the west to the interior in the east, remotely-sensed data shows that there are gradients in climate and vegetation along this axis, with the country being warmer and wetter in the east. Land surface temperatures and values of seasonally peaked precipitation, a measure of the proportion of the point’s annual rainfall that falls in the wettest 91-day period, were both highest in the east. Warm and humid conditions provide ideal conditions for adult mosquitoes to thrive and contribute to the high vector survival in the east of the country. Nationally, increased vector survival was not only associated with warm and wet conditions, it was also associated with proportionately fewer metal-roof houses. Similarly, in the study carried out in eastern Gambia, mosquito survival within individual villages was associated only with the percentage of metal-roof houses and not climate covariates. While all of The Gambia is suitable habitat for *Anopheles* from a climatic perspective, the eastern area has persistent (and higher) malaria transmission suggesting that the climatic conditions in this area provide better vector habitat than the rest of the country. However, we found that residual transmission within The Gambia was correlated with metal roof density and not coarse-scale climatic factors. This does not imply that climate and malaria are decoupled, but rather than the current, localized distribution of falciparum malaria is a product of numerous factors including climatic factors, historical malaria burden, historical and present intervention coverage, patterns of treatment seeking and healthcare access, and, as this study suggests, metrics of housing quality.

Our hypothesis is based on the principle that the main malaria vectors in The Gambia are endophilic and endophagic, namely resting and feeding and indoors for several days before exiting to lay eggs. Indeed, in a previous study, only 0.6% of blood-fed mosquitoes exited from houses during the night with untreated nets and 6.4% did so when houses had insecticide-treated nets^31^. Movement of blood-fed *An. gambiae s.s*. between houses occurs infrequently, and it is common that mosquitoes feeding outdoors will rest indoors^32^. In theory, this enables indoor vector control interventions such as metal roofs to reduce malaria transmission by lowering the survival of blood-fed mosquitoes.

Although metal-roof houses may be viewed as reducing vector survival, more evidence may be needed to support their uptake on a large scale for malaria control. They are not an evolution-proof intervention since an increased mortality of indoor-resting mosquitoes may select for exophily and exophagy^33^, with malaria vectors being selected to rest and bite outdoors. In addition, it is possible that there may be unintended consequences of different roof materials. For example, reduced ventilation may increase the indoor concentration of human host odours and subsequent attractiveness of the home to vectors through any openings^23^. Finally, the increased temperatures associated with metal-roofs are less comfortable for people in the daytime and may increase the number of people sleeping outdoors during hot nights. Nonetheless, our study highlights the potential of metal roofs to reduce vector survival, which may contribute to a community impact on malaria transmission. At a time when malaria control has stalled in sub-Saharan Africa^34^, there is a real need to develop supplementary interventions, like improved housing, against this lethal disease.

There are several limitations associated with this study. First, the daily survival recorded from the caged experiments in village houses are probably higher than those experienced by wild mosquitoes because the laboratory mosquitoes reared under constant temperatures, may have been stressed by the fluctuating temperatures experienced in village houses and/or damaged during transport from the laboratory to the villages by vehicles traveling along bumpy roads. Thus the survival rates in the different house typologies should not be interpreted literally, but comparatively. Second, the cages in the village houses prevented mosquito movement, so our experiment did not replicate the movement from the roof to the floor seen with wild mosquitoes. Third, our laboratory studies were carried out at constant temperatures and the longevity of adult mosquitoes may have been underestimated^35^. Finally, metal roofs are likely to be associated with increased wealth, which can help reduce malaria risk through other mechanisms such as increased access to healthcare, although wealth would not affect mosquito parity.

## Conclusion

Here we provide support for the hypothesis that the large-scale introduction of metal-roof housing may have a mass-killing effect on *An. gambiae* mosquitoes resting indoors in hot climates in sub-Saharan Africa. Indoor temperatures approaching 35 °C are more common in metal-roof houses than thatched houses and result in higher vector mortality. Such rises in temperature are also likely to prevent the development of malaria parasites in vectors^29^. It is possible that the mass killing of vectors by metal-roofs may be one factor contributing to the low levels of malaria transmission experienced in urban areas, which are approximately 80% lower than in rural areas^36^. Moreover, our findings are consistent with the hypothesis that changes to the African housing stock may explain part of the malaria decline observed in sub-Saharan Africa between 2000 and 2015 that is not attributable to health interventions^1^. The potential impact of development and changes to the African housing stock should be considered by those interested in measuring the future impact of environmental change on malaria transmission in sub-Saharan Africa, particularly in respect of climate change.

## Methods

### Study area

Field studies were carried out in rural Gambia in the Central River Region (CRR) and in the Upper River Region (URR), in the extreme east of the country. The country experiences a single rainy season from June to October, followed by a long dry season. Most malaria cases occur between September and December.

### Mosquito survival in metal- and thatched-roof houses

Survival of blood-fed female *An. coluzzii* was compared in single roomed ventilated metal-roof houses and thatched-roof houses in 2016 and between unventilated metal-roof houses and thatched-roof houses in 2017.

In 2016, we selected 15 villages closest to the MRCG insectary at Basse Santa Su that were in the Roo*Pf*s Study^22^ and had at least three houses in each study group (ventilated-metal and thatched roof houses). Eligible houses were then randomized using statistical software (Stata version 14) to provide a list of two first choice and, in case of drop out, one second choice house, in each study group in each village. In 2017, three randomized thatch houses were selected, two as first choice and one a second choice in each village. Two thatched houses and the nearest metal-roof house in the same compound as each was selected as the comparator.

Both experiments were carried out during the rainy season in 15 study villages close to an insectary at Basse Santa Su (13°18′30″North. 14°12′55″West). Houses were enrolled from villages in a study designed to measure the impact of improved housing on malaria^22^. Study houses were randomized to receive metal-roofs, closed eaves, a screened window in the gable ends and screened doors to encourage ventilation in the treatment arm (referred to as ventilated metal-roofs) or remained with thatched-roofs, open eaves and no screening in the control arm (Fig. 1). Pairs of metal- and thatched-roof houses were randomly selected from the same compound. In 2016, the comparison was made between ventilated metal-roof houses and thatched houses, whilst in 2017 the comparison was between typical metal-roof houses (hereafter referred to as unventilated houses) and thatched houses. Written informed consent from the household head was obtained for these additional studies before starting the experiments. Each week a metal-roofed house and thatched-roofed house in the same compound were selected randomly, with two pairs of houses in the same village.

200–300 insectary-bred, unfed, two to five-day old, adult female *An. coluzzii* (Yaoundé strain) were starved of sugar water for 24 h and fed on rabbits or humans. Groups of 30 blood-fed mosquitoes were placed in holding cups and transported by car to the study villages. In each village, 30 mosquitoes were released into a cylindrical-shaped cage, 48 cm high made from solid polyester with PVC coating, with cotton netting tops and bottoms 45 cm in diameter (modified Garden Bag – 150 L, Homebase, UK). Each cage was placed with the bottom 1–1.5 m above the floor, close to a “Jibida” (a traditional clay water storage jar), where mosquitoes commonly rest, before midday. In 2016, mosquitoes were provided with cotton wool soaked in 10% sugar solution and placed on top of the cage with a petri-dish covering it. If the cotton pad was dry at the end of day one it was wetted again. In 2017, no sugar solution was provided. Mosquito survival was recorded after 24 h and 48 h. Temperature and relative humidity was recorded every 30 minutes using a data logger (Tinytag Ultra2, Gemini data loggers, UK) placed inside the cage when the mosquitoes were released.

### Mosquito behaviour in metal-roof houses

Movement of wild *An. gambiae* s.l. indoors during the day was recorded in single-roomed, metal-roof houses with closed eaves in two villages; three houses in Saruja (13°33′0″North, 14°55′0″West) and two houses in Wellingara (13°38′0″North, 15°2′0″West) in May 2003, at the end of the dry season. After obtaining permission from the household head, indoor-resting mosquitoes were collected from five metal-roof houses with an aspirator over three consecutive days. One or two people slept under untreated bednets in each room. At 07.00 h houses were searched for mosquitoes using a torch and an aspirator for 30 person minutes. The roof was searched for first 15 minutes, approximately 2.4 m above the floor, whilst the second 15-minute collection was restricted to mosquitoes found less than 0.6 m from the floor; usually from the floor itself, under the bed or around the water pot. Temperature was recorded using a digital thermometer (Probe type T, Digitron Sifam Instruments Ltd, Torquay, UK) close to the roof surface and near the floor, both points where mosquitoes were caught that day. Doors and windows were kept closed to minimise mosquito disturbance. Houses were revisited at 14.30 h and repeat collections made from the roof and walls. Mosquitoes were sorted to species and *An. gambiae* s.l. identified by PCR^37^.

### Survival of *An. gambiae* adults in the laboratory

The 16CSS strain of *An. gambiae s.s*. was maintained at 26 °C ± 1 °C and 80% relative humidity in a laboratory at Durham University, UK in 2000. Eggs were hatched at 25–26 °C and the larvae fed fish food (TetraMin fish flakes, Tetra, UK). Adult mosquito mortality was measured for combinations of temperatures 20–40 °C at 5 °C intervals and humidities 40–100% at 20% intervals. For each combination of temperature and humidity, four sets of replicate tests were performed. Pupae were collected daily and allowed to hatch at 26 °C in colony cages (30 × 30 × 30 cm). On the second day of eclosion, 50 adults, 20 males and 30 females, were moved from colony cages into test cages of 15 × 15 × 15 cm housed in rectangular glass chambers (39 × 20 × 22 cm). Each cage held one sugar fountain with 10% glucose solution and was topped-up daily, with the lint wick from the drinking fountain changed weekly. The glass chambers were housed in environmental cabinets (Cooled Incubators, LMS Ltd, Kent, UK) at the requisite temperature. To maintain 100% relative humidity, a bowl of distilled water was placed in the glass chamber and for lower humidities a range of concentrations of potassium hydroxide solution was used^38^. Humidity and temperature within each glass chamber was monitored using loggers (Tiny Talk II, Gemini, UK), and the solutions replaced whenever the humidity readings fell outside ±10%. Each chamber was adjusted to the appropriate constant temperature (±0.5 °C) and a 12:12 h light and dark regime. The number of dead mosquitoes was recorded daily and each dead mosquito removed from the cage. Each day, chambers were removed from the cabinet and the top opened for about one minute to replenish the air inside the chamber. Survival analysis was confined to only female mosquitoes.

### Malaria surveys

The malaria surveys are described in detail elsewhere^20,39^. Briefly, two longitudinal entomological and epidemiological studies were carried out: one nationwide and one in the Upper River Region (URR), the region with the highest malaria transmission. We selected the years in which the largest numbers of *An. gambiae* s.l. were dissected for parity. In the nationwide study, six pairs of villages across The Gambia (West Coast Region, North Bank Region, Lower River Region, CRR, URR north bank and the URR south bank) were included. Each pair of villages were one to three kilometres apart, with populations of 100–700 people. After obtaining informed consent, all residents aged from six months to 15 years old had monthly blood samples collected by finger-prick, from June to December 2013, for malaria diagnosis by nested PCR^40^. Monthly entomological sampling was done using human-landing catches (HLCs) from 19.00 h to 07.00 h. Indoor and outdoor HLCs were made in two houses per village for three consecutive nights monthly, from June to December 2013. Mosquitoes were counted and species identified morphologically by PCR^37^. *Anopheles gambiae s.l*. females captured by HLCs were dissected to remove the ovaries and determine parity^41^.

The type of roofs in each study village was determined by field teams. Data on climate, surface water and land cover were collected for each village using remotely sensed monthly data^42^ over the wet season, from June to November 2013. The remotely-sensed data included: (1) enhanced vegetation index (EVI), a measure of the proportion of photosynthetically active radiation absorbed by vegetation, which is correlated with vegetation density and active photosynthesis, (2) day and night land surface temperature (LST), strongly correlated with air temperature, (3) the diurnal difference in LST, a proxy for humidity, (4) tasselled cap wetness (TCW), a proxy for moisture in the environment, (5) tasselled cap brightness (TCB), a proxy for dry, bare soil and senescent vegetation, (6) seasonally peaked precipitation (SPP), the fraction of the point’s annual precipitation that falls within the wettest 91 day period, rescaled for the whole continent with values from zero to one (high values correspond to a narrower rainy season window and/or a more peaked rain pulse relative to the rest of the year), (7) synoptic precipitation (SP) derived from the WorldClim surface from 1980–2010 data^43^, (8) proportion of ephemeral water within 1 or 2 km from the JRC landcover surface^44^, (9) proportion of forest within 1 and 2 km buffers in year 2000 as extracted from the Hansen global forest cover dataset^45^, and the proportion of urban area within 1 and 2 km using the Global Urban Footprint dataset (DLR Earth Observation Center).

In the regional survey in the URR, 70 clusters of one to three villages were enrolled as part of a trial to measure the efficacy of LLINs and IRS^39^. Approximately 110 children aged 6 months to 14 years old were enrolled from each cluster and finger-prick samples taken at the end of the transmission season in January 2011. Thick blood films were stained with Giemsa and examined for malaria parasites by microscopy. In 32 clusters, mosquitoes were collected using CDC light traps indoors from six sentinel houses where at least one adult slept under a LLIN. Sampling was done monthly, from July to December 2010. Mosquitoes were counted and species identified morphologically and by PCR^37^. *Anopheles gambiae s.l*. females were dissected to extract the ovaries and measure parity^41^. Household surveys collected data on roof type for all village houses. Environmental data were collected as described above covering the period June to December 2010.

### Data analysis

Paired t-tests were used to examine differences in temperature and vector mortality in pairs of thatched-roof houses and metal-roof houses in each village and between mosquito collections made on the roof and floor of village houses. Linear regression was used to explore the relationship between mosquito survival in the laboratory and temperature, humidity and time.

In the nationwide study, the association between roof type (metal *versus* thatch) and remotely-sensed environmental variables with vectorial capacity and mosquito parity and malaria parasite rates among children was explored among villages. Vectorial capacity, *V*, is a measure of malaria transmission risk and is defined as the number of infective bites that could originate from an infective individual expressed as a daily rate^24,46^. *V* was estimated for each village using the following equation^47^:

$$V=\frac{m{a}^{2}\,{p}^{n}}{-{\mathrm{log}}_{e}p}$$

where *m* is the mean number of *An. gambiae* s.l. per person, *a* is the human-biting habit derived by multiplying the proportion of mosquitoes feeding on people (human blood index, HBI) by the feeding frequency in days, *r*, which was assumed to be once every two days (i.e. *r* = 0.5) biting per night collected from human-landing. More simply *ma* is considered equivalent to the mean number of *An. gambiae* s.l. collected per individual during human landing catches. The HBI was assumed to be the same as previously recorded in The Gambia during the rainy season^26^. The probability of a mosquito surviving one day, *p*, was determined from parity using the equation^48^:

$$p={A}^{1/x}$$

where *x* is the interval between blood meals, in this case two days, and *A* is the proportion of parous mosquitoes. The extrinsic period of development of the parasite (*n*) is the number of days taken for the parasite to become infective in the mosquito, assumed to be 10, the minimum period required for the malaria parasite to become infective in the vector minimum value^24^. Associations between environmental variables and entomological and clinical outcomes were explored using linear regression. Statistical analysis used SPSS version 22.

### Ethical considerations

Approval for this work was given by the Gambian Government and MRCG Unit Joint Ethics Committee and the Department of Biological and Biomedical Science’s ethics committee of Durham University. All methods were performed in accordance with the relevant guidelines and regulations. Informed consent was obtained from all study participants.

## Supplementary information

Supplementary material

## Data Availability

The data are available from the corresponding upon reasonable request.
