# Supplementary material for: Reduced mosquito survival in metal-roof houses may contribute to a decline in malaria transmission in sub-Saharan Africa
Source: Sci Rep. 2019 May 23;9:7770. doi: 10.1038/s41598-019-43816-0 (PMC6533302; doi:10.1038/s41598-019-43816-0)
Supplement: Supplementary file 1 — Supplementary material [file 41598_2019_43816_MOESM1_ESM.docx]

**Supplementary material**

**Table 1.** Rate ratios of mosquito mortality at different combinations of temperature and relative humidity. Where CI are confidence intervals.

| Variable | Rate ratio (95% CI) | z | P |
| --- | --- | --- | --- |
| Temperature (^o^C) | | | |
| 20 | 1.0 |  |  |
| 25 | 1.14  (1.04-1.26) | 2.67 | 0.008 |
| 30 | 2.00  (1.82-2.21) | 13.86 | <0.001 |
| 35 | 4.05  (3.67-4.47) | 27.90 | <0.001 |
| 40 | 24.81  (22.49-27.37) | 64.12 | <0.001 |
| Relative humidity (%) | | | |
| 40 | 1.0 |  |  |
| 60 | 0.85  (0.78-0.93) | -3.58 | <0.001 |
| 80 | 0.75  (0.68-0.82) | -6.50 | <0.001 |
| 100 | 0.82  (0.75-0.90) | -4.34 | <0.001 |

**Supplementary material**

**Table 2.** Calculation of vectorial capacity in the nationwide study villages.

| Village | Region | % metal roofs | *ma* | HBI^26^ | *r* | *a* | *A* | *x* | *p* | *n* | *V* | Parasite prevalence |
| --- | --- | --- | --- | --- | --- | --- | --- | --- | --- | --- | --- | --- |
| Bessi | WCR | 100.0 | 12.2 | 0.705 | 0.5 | 0.35 | 0.392 | 2 | 0.626 | 10 | 0.0844 | 4.68 |
| Ndemban | WCR | 100.0 | 3.86 | 0.705 | 0.5 | 0.35 | 0.500 | 2 | 0.707 | 10 | 0.1218 | 2.56 |
| Chogen | NBR | 81.0 | 1.18 | 0.705 | 0.5 | 0.35 | 0.323 | 2 | 0.568 | 10 | 0.0026 | 4.29 |
| Yallal | NBR | 76.7 | 1.25 | 0.705 | 0.5 | 0.35 | 0.216 | 2 | 0.465 | 10 | 0.0003 | 3.00 |
| Sinchu Njengudi | LRR-S | 100.0 | 12.7 | 0.705 | 0.5 | 0.35 | 0.479 | 2 | 0.692 | 10 | 0.3046 | 3.38 |
| Dongoro Ba | LRR-S | 98.6 | 3.06 | 0.705 | 0.5 | 0.35 | 0.567 | 2 | 0.753 | 10 | 0.2212 | 10.77 |
| Sare Seedy | CRR-N | 41.5 | 5.58 | 0.705 | 0.5 | 0.35 | 0.676 | 2 | 0.822 | 10 | 1.4082 | 2.53 |
| Ngedden | CRR-N | 31.3 | 5.73 | 0.705 | 0.5 | 0.35 | 0.669 | 2 | 0.818 | 10 | 1.3372 | 1.39 |
| Njayel | URR-S | 44.8 | 2.37 | 0.705 | 0.5 | 0.35 | 0.968 | 2 | 0.984 | 10 | 43.354 | 10.45 |
| Madina Samako | URR-S | 76.0 | 1.96 | 0.705 | 0.5 | 0.35 | 0.975 | 2 | 0.987 | 10 | 47.748 | 22.86 |
| Sare Wuro | URR-N | 15.4 | 2.9 | 0.705 | 0.5 | 0.35 | 0.921 | 2 | 0.960 | 10 | 16.346 | 21.00 |
| Gunjur Koto | URR-N | 30.0 | 2.05 | 0.705 | 0.5 | 0.35 | 0.979 | 2 | 0.989 | 10 | 60.806 | 30.72 |

Where, WCR is West Coast Region, NBR is North Bank Region, LRR is Lower River Region, CRR is Central River Region (CRR), and URR-S is Upper River Region south bank and URR-N is URR north bank, *ma* is the mean number of *An. gambiae s.l.* biting per night, HBI is the human-biting index, *r* is the feeding frequency, *a* is the human-biting habit, *A* is the proportion of parous mosquitoes, *x* is the interval between blood meals, *p* is the probability of a mosquito surviving one day, *n* is the extrinsic incubation period of the parasite, *V* is vectorial capacity and parasite prevalence is the total number of *Plasmodium falciparum* infections for children aged 6 months to 15 years old during the monthly surveys from June to December 2013 detected by PCR.
